# Crack Propagation of Ceramsite Lightweight Concrete Under Four-Point Bending Fatigue Conditions

**DOI:** 10.3390/ma18132957

**Published:** 2025-06-22

**Authors:** Kangqing Yang, Shenghan Zhuang, Yongjun Wang, Jiashu Li, Shuo Zhou, Jiaolong Ren

**Affiliations:** 1 School of Civil Engineering and Geomatics, Shandong University of Technology, Zibo 255000, China; 22110902065@stumail.sdut.edu.cn (K.Y.); 23507030871@stumail.sdut.edu.cn (Y.W.); 22110902003@stumail.sdut.edu.cn (J.L.); 22110902175@stumail.sdut.edu.cn (S.Z.); 2 Department of Bridge Engineering, Southwest Jiaotong University, Chengdu 610031, China; shenghanzhuang@my.swjtu.edu.cn

**Keywords:** ceramsite lightweight concrete, fatigue crack, crack location, crack angle, ceramsite size, ceramsite content

## Abstract

The examination of crack propagation in concrete under fatigue conditions is crucial for comprehending the mechanisms of concrete fatigue failure. Variations in aggregate types result in notable differences in the fatigue crack propagation characteristics of lightweight concrete compared to ordinary concrete. Consequently, this research focused on analyzing the locations and angles of cracks in ceramsite lightweight concrete subjected to four-point bending fatigue conditions, while accounting for different levels of fatigue loading (i.e., stress levels). Furthermore, the study aimed to clarify the influence of ceramsite size and content on the fatigue crack propagation behavior in ceramsite lightweight concrete. The results indicated that an increase in the replacement rate of 5–10 mm and 10–20 mm ceramsite led to the highest probability of fatigue cracks occurring within the range of 15–45 mm from the specimen center, reaching 41.2% and 44.7%, respectively. The crack angle exhibited an increase corresponding to an increase in the content of 5–10 mm ceramsite, with the maximum average crack angle attaining a value of 86.5°. Conversely, a decrease in the content of 10–20 mm ceramsite resulted in a reduction in the crack angle. However, 20–30 mm ceramsite did not have a significant effect on the characteristics of fatigue cracks. The level of stress predominantly influenced the path of crack propagation. At stress levels of 0.55, 0.65, and 0.75, the highest proportions of crack angles fell within the range of 75° to 80°, with values of 47.1%, 43.8%, and 53.3%, respectively. Furthermore, an increase in stress levels did not affect the location of the cracks.

## 1. Introduction

In recent years, lightweight concrete has emerged as a prominent material in the construction of high-rise buildings and large-span structures, owing to its notable attributes, including high strength, reduced weight, enhanced durability, and environmental sustainability [1,2,3,4,5,6]. The fundamental difference between lightweight concrete and traditional cement concrete lies in the use of various lightweight aggregates, such as ceramsites [7], slag [8], and sand [9]. The beneficial characteristics of lightweight aggregates, which include low density, superior thermal insulation properties, strong seismic resistance, and well-established industrial production methods, have been effectively leveraged in the application of lightweight concrete.

Furthermore, modern engineering structures are vulnerable to the impacts of prolonged cyclic loading, which may lead to a decline in their performance over time, thereby diminishing their service life and increasing the risk of fatigue failure [10,11,12,13]. Consequently, investigating concrete fatigue failure is of paramount importance for ensuring structural safety and facilitating consistent operational stability [14]. The mechanisms underlying the formation of fatigue cracks are attributed to the phenomenon of stress concentration that occurs in vulnerable regions, such as initial voids, within a concrete specimen when subjected to fatigue loading. This concentration of stress promotes the initiation of fatigue cracks in these vulnerable areas of the concrete [15]. Furthermore, under prolonged fatigue loading conditions and with the intrinsic non-uniformity of concrete, a significant proportion of the developed fatigue cracks are inclined to propagate along the peripheries of the aggregates. This propagation ultimately culminates in the fatigue failure of the concrete [16,17]. Consequently, investigating fatigue cracks in concrete is of significant importance. Liu et al. [18] demonstrated that monitoring crack progression in concrete through acoustic emission techniques can function as an early warning system for potential structural failures. In a similar vein, Zhao et al. [19] developed a theoretical model for predicting fatigue cracks, which allows for the estimation of the residual fatigue life of concrete. Additionally, Miura et al. [20] showed that the existence of primary cracks can lead to a reduction in the compressive strength of concrete and subsequently shorten its fatigue lifespan. Collectively, these studies highlighted the essential role of fatigue cracks in the evaluation and enhancement of the fatigue and structural performance of concrete. Moreover, several constitutive models have also been established to describe the fatigue crack of concrete. For instance, the Concrete Damage Plasticity (CDP) model operates under the assumption that concrete is a homogeneous material, thereby simplifying the inherently heterogeneous nature of concrete. Consequently, this model could simulate the main crack more efficiently, but failed to accurately represent the crack propagation caused by aggregate inhomogeneous distribution [21]. Additionally, the Cohesive Zone Model (CZM) necessitates the determination of the initial crack at two specific points adjacent to the crack path; however, the selection of these points depends on the distribution of mesoscopic structures, which hinders the ability of the CZM to realistically simulate cracking behavior [22].

The properties of aggregates are crucial for the investigation of the fatigue performance of concrete [23,24,25,26,27,28]. This is particularly relevant for lightweight aggregate concrete, where the properties of the aggregates significantly influence the formation of fatigue cracks. Research conducted by Li et al. [29] indicated that the incorporation of ceramsites, in terms of both their quantity and size, can improve the fatigue performance of concrete containing ceramic particles. Similarly, Lv et al. [30] demonstrated that the properties of rubber particles can prolong the fatigue life and diminish the fatigue strain in self-compacting rubber lightweight aggregate concrete. Additionally, Aslam et al. [31] discovered that the addition of oil-palm-blower clinkers substantially enhanced the elastic modulus of lightweight aggregate concrete. It is clear that the characteristics of aggregates are critically important for understanding the mechanisms of fatigue failure in lightweight aggregate concrete.

Despite the existing research, there remains a significant gap in the literature concerning the influence of aggregate characteristics on fatigue cracking in lightweight concrete. Notably, Cui et al. [32] found that the incorporation of mixed fibers in high-strength lightweight aggregate concrete reduced the rate of crack propagation, and they proposed a mathematical expression to characterize the relationship between crack propagation rates and crack lengths under varying stress conditions. Furthermore, Zhang et al. [33] reported that the addition of mechanism sand to self-compacting lightweight aggregate concrete resulted in a higher incidence of secondary cracks and a more uniform distribution of cracks, which facilitated the determination of an optimal dosage of mechanism sand for this type of concrete. However, the studies conducted thus far have largely overlooked the effects, underlying mechanisms, and patterns related to the characteristics of lightweight aggregates regarding fatigue crack propagation in lightweight concrete.

Hence, ceramsite lightweight concrete was utilized as the primary subject of investigation. Four-point bending fatigue tests were performed to examine the fatigue life and crack development in lightweight concrete incorporating ceramsite aggregates of varying sizes (5–10 mm, 10–20 mm, and 20–30 mm) and subjected to different levels of fatigue loading. Additionally, the study elucidated the influence of both the size and content of ceramsite aggregates on the propagation of fatigue cracks within ceramsite lightweight concrete.

## 2. Materials and Experimental Methods

### 2.1. Material

(1) Cement

Ordinary Portland cement P.O. 42.5 was utilized in this study, and its technical properties are presented in Table 1.

(2) Aggregates

**Figure 1:**
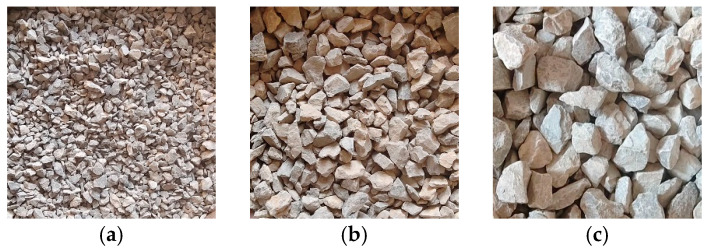
Aggregates with different particle sizes. (**a**) 5–10 mm, (**b**) 10–20 mm, and (**c**) 20–30 mm.

Basalt from Zibo, Shandong, was used as the aggregate in this study, which was divided into three sizes: 5–10 mm, 10–20 mm, and 20–30 mm, as illustrated in Figure 1. The technical properties of the aggregate are listed in Table 2.

(3) Ceramsites

**Figure 2:**
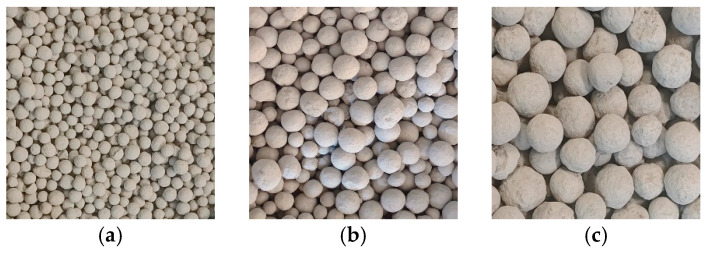
Ceramsites with different sizes. (**a**) 5–10 mm, (**b**) 10–20 mm, and (**c**) 20–30 mm.

Ceramsites produced by Zhejiang Ningbo Zhongjin Environmental Protection Technology Co., Ltd. in Ningbo City, Zhejiang Province, China, were used in this study, as shown in Figure 2. Their particle sizes were divided into three sizes: 20–30 mm (designated as CRR1), 10–20 mm (designated as CRR2), and 5–10 mm (designated as CRR3), with packing densities of 920 kg·m^−3^ (CRR1), 1000 kg·m^−3^ (CRR2), and 970 kg·m^−3^ (CRR3), respectively. The technical properties are presented in Table 3.

(4) Silica fume

Silica fume produced by Hebei Boheng Mining Co., Ltd. was selected. Its technical properties are shown in Table 4.

(5) Fly ash

Grade II fly ash produced by Hebei Boheng Mining Co., Ltd. was used in this study. Its technical properties are shown in Table 5.

(6) Sand

River sand was selected as the fine aggregate in this study, with a fineness modulus of 2.7. Its technical properties are shown in Table 6.

(7) Water-reducing agent

A polycarboxylate superplasticizer produced by Hongxiang Building Additive Factory in Laiyang City, Shandong Province, China, was utilized in this study, with a bulk density of 600 ± 100 g/L. Its technical properties are presented in Table 7.

### 2.2. Mix Proportion

According to the Chinese standard “Specification for Mix Proportion Design of Ordinary Concrete (JGJ 55-2011)” [34], the specified water–cement ratio was 0.28, the sand ratio was 0.36, and the water-reducing agent was incorporated at a level of 2% of the weight of the cement. Additionally, fly ash and silica fume were utilized as partial replacements for cement, at proportions of 20% and 10%, respectively.

This research entailed the creation of 12 experimental sets, which were designed by modifying the replacement rates of ceramsites of different sizes: 20–30 mm, 10–20 mm, and 5–10 mm. It was important that the substitution of natural aggregates with ceramsite was predicated on the principle of equal volume replacement. To minimize calculation inaccuracies, three varieties of ceramsite that matched the sizes of the natural aggregates were utilized. Previous research [35,36,37] indicates that the ceramsite replacement rate should not exceed 40%. In light of these findings, the present study established ceramsite replacement rates of 10, 20, 30, and 40%, as outlined in Table 8. Furthermore, due to the larger size of the 20–30 mm ceramsite, the quantity of this material was limited at lower replacement rates, making it challenging to ensure effective distribution of the ceramsite. Therefore, the replacement rates for the 20–30 mm ceramsite were designated at 20, 30, 40, and 50%.

### 2.3. Experimental Methods

#### 2.3.1. Preparation of Test Specimen

**Figure 3:**
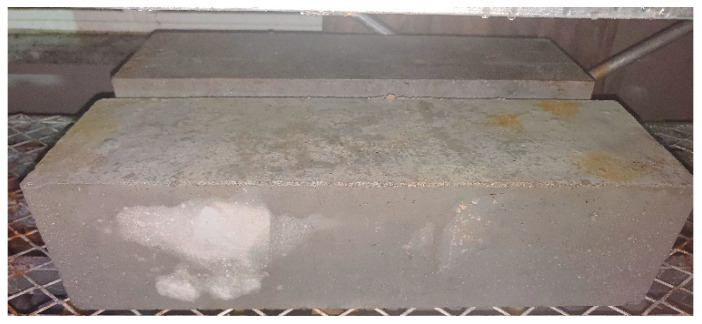
Standard curing process of specimens.

According to the Chinese standard “Standard Test Method for Performance of Ordinary Concrete Mixtures (GB/T 50080-2016)” [38], the dimensions of the rectangular beam specimens prepared were length × width × height = 550 mm × 150 mm × 150 mm, with a curing period of 90 days, as shown in Figure 3.

#### 2.3.2. Fatigue Test

**Figure 4:**
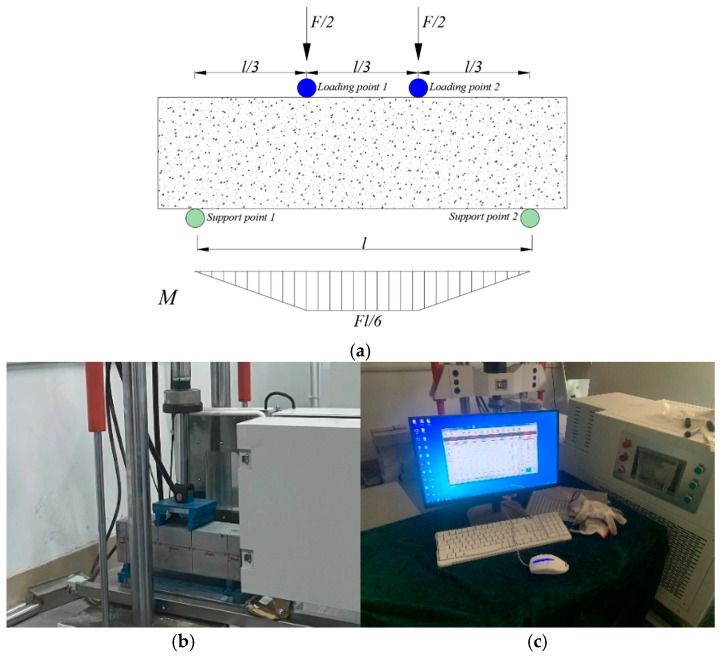
Schematic diagram of fatigue test. (**a**) Four-point fatigue test force and bending moment situation, (**b**) testing machine, (**c**) and test machine control interface.

A four-point bending fatigue experiment for ceramsite concrete was adopted in this study, as shown in Figure 4.

The parameters of the fatigue test are shown in Table 9.

It is essential to note that the loading frequency for concrete fatigue tests is typically determined within the range of 4 to 10 Hz, as stipulated by Chinese testing standards [39,40]. In the present study, the loading frequency was established at 10 Hz, considering the operational capabilities of the fatigue testing apparatus employed. Additionally, prior research [41,42,43] suggests that stress levels usually fall between 0.5 and 0.9. For the purposes of this investigation, the stress levels were designated at 0.55, 0.65, 0.75, and 0.85 as a precautionary measure. Lastly, the characteristic value of the load cycle, defined as the stress ratio, and the loading speed were set at 0.1 and 0.005 mm/s, respectively, to maintain the stability of the specimen throughout the bending fatigue test, in accordance with the recommendations provided by the instrument manufacturer.

Static four-point bending tests were performed to assess the bending strength of ceramsite lightweight concrete prior to the execution of fatigue testing. Following this, the stress levels were computed in accordance with Equation (1) to define the parameters for fatigue loading. In the present study, the designated stress levels were established at 0.55, 0.65, 0.75, and 0.85.

(1) $$S=\frac{\sigma_{\max}}{f_{r}}$$

where *S* is the stress level; *σ*_max_ is the fatigue peak stress; and *f_r_* is the bending strength under static loading.

**Figure 5:**
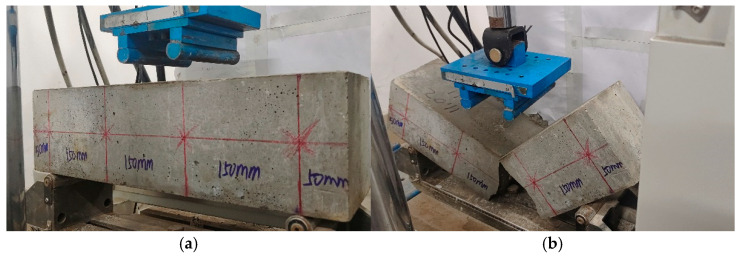
Specimen placement and fracture. (**a**) Test piece placement, (**b**) test piece fracture.

The fatigue test process was as follows:

- Prior to the formal loading process, it was necessary to conduct preloading in order to eliminate any discrepancies between the contact surfaces of the test beam and the testing apparatus, thereby facilitating accurate data collection. The preloading load was set at 20% of the maximum limit of the fatigue test load. The method for specimen placement is illustrated in Figure 5a.
- Further stress levels *S* of 0.55, 0.65, 0.75, and 0.85 were selected for the four-point bending fatigue test of ceramsite concrete, with loading until the specimen fractured. The bending strength can be determined using Equation (2). A fractured specimen is shown in Figure 5b.

(2) $$f_{t}=\frac{Fl}{bh^{2}}=\frac{Fl}{150\times 150^{2}}$$

**Figure 6:**
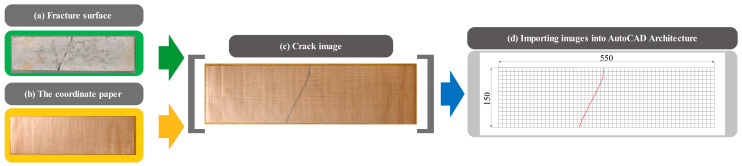
Process for recording crack locations and angles.

The following process was implemented to record the crack locations and angles of failure specimens after fatigue testing, as illustrated in Figure 6.

- Position the coordinate paper on either side of the specimen exhibiting failure and meticulously outline the cracks on the coordinate paper, as illustrated in Figure 6a–c.
- Obtain a photograph of the coordinate paper to generate an image depicting the cracks, and subsequently import this image of the cracks into AutoCAD Architecture 2024, as illustrated in Figure 6d.
- Utilizing AutoCAD Architecture, one can construct a coordinate grid, align the image with the coordinate axes, and subsequently identify and export the coordinates of the cracks, using the commands “REC”, “AL”, and “DIMORD” respectively.

By obtaining the coordinates of certain feature points for each crack, such as the initial point, terminal point, and inflection point, the AutoCAD Architecture is subsequently utilized to connect these coordinates, thereby creating a crack. Following this, the “DIMANGULAR” command can be employed to determine the angle of this crack.

Images of concrete cracks are captured using high-definition cameras that provide adequate image resolution. These images are then imported into AutoCAD Architecture using the “ATTACH” command, which preserves the original image resolution. Additionally, since the dimensions of the crack images may vary upon importation into AutoCAD Architecture, a coordinate grid with identical dimensions at a 1:1 scale is established within the software prior to the import process. Afterward, the crack image is scaled to align with the pre-drawn coordinate grid, thereby maintaining the original proportions of the crack image.

## 3. Analysis of Fatigue Crack Characteristics

### 3.1. Fatigue Test Results

**Figure 7:**
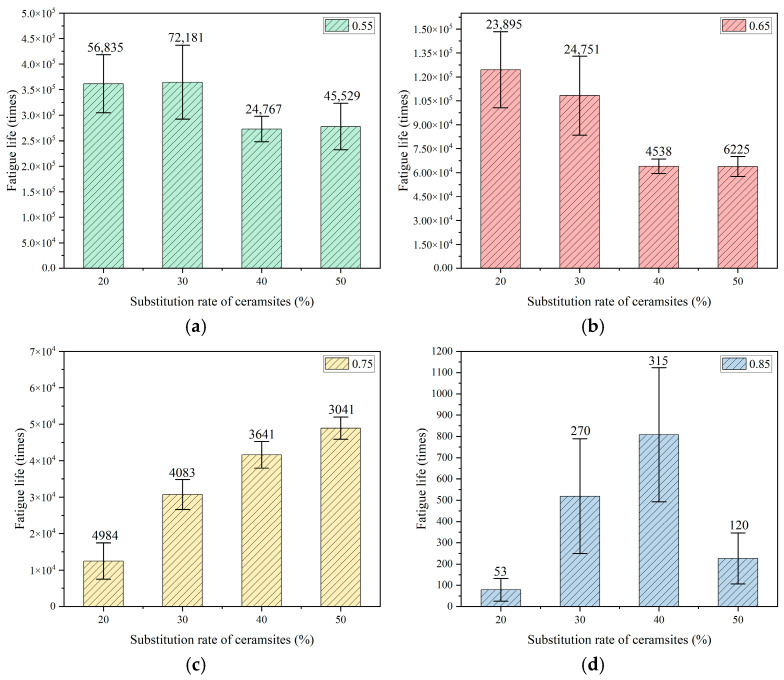
The effect of a 20–30 mm ceramsite replacement rate on fatigue life. (**a**) The stress level was 0.55, (**b**) the stress level was 0.65, (**c**) the stress level was 0.75, (**d**) the stress level was 0.85.

**Figure 8:**
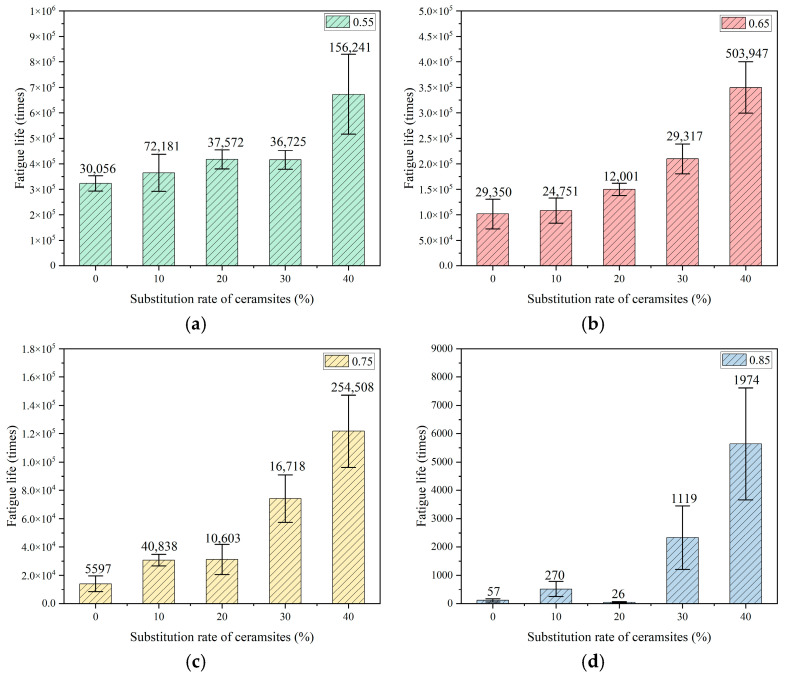
The effect of a 10–20 mm ceramsite replacement rate on fatigue life. (**a**) The stress level was 0.55, (**b**) the stress level was 0.65, (**c**) the stress level was 0.75, (**d**) the stress level was 0.85.

**Figure 9:**
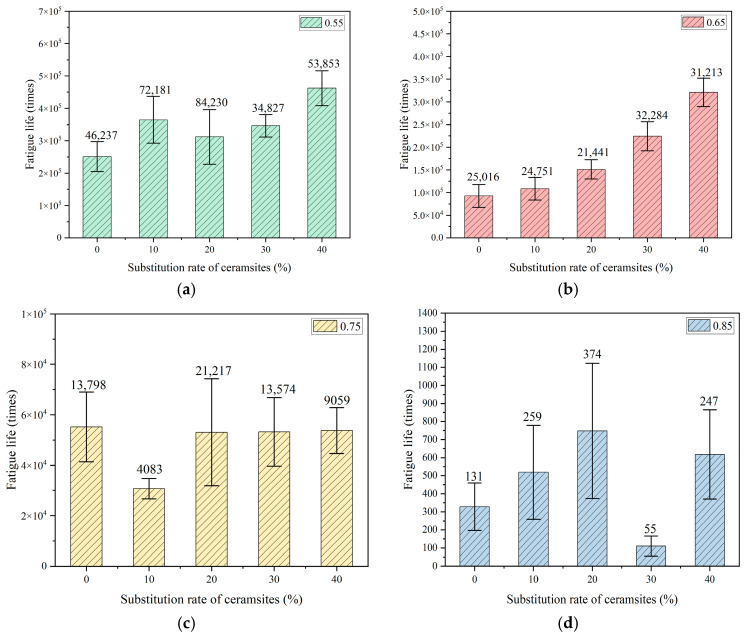
The effect of a 5–10 mm ceramsite replacement rate on fatigue life. (**a**) The stress level was 0.55, (**b**) the stress level was 0.65, (**c**) the stress level was 0.75, (**d**) the stress level was 0.85.

To reduce the experimental error associated with the intrinsic variability of fatigue testing, six parallel specimens were evaluated within each group. The mean value for each group was derived from the gathered fatigue data. The relationships established between the replacement rate of ceramsites in lightweight aggregate concrete and fatigue life, as well as the correlation between stress level and fatigue life, are illustrated in Figure 7, Figure 8 and Figure 9.

In accordance with Chinese standards [39,40,44], it is mandated that a minimum of three specimens be employed for fatigue testing. This investigation successfully measured six specimens. Additionally, these standards specify a reliability criterion for fatigue life data, which stipulates that the deviation of any measured value from the mean must not exceed 1.82 times the standard deviation when utilizing six valid specimens. The fatigue lives illustrated in Figure 7, Figure 8 and Figure 9 in this study conform to these established standards, thereby affirming the reliability of the fatigue life data presented.

A variance analysis of fatigue life was performed, as presented in Table 10, Table 11 and Table 12. SSB and SSW represent the sum of squares between groups and the sum of squares within groups, respectively. The results indicate that all *p*-values were below the threshold of 0.05, suggesting that the data pertaining to fatigue life were statistically significant.

### 3.2. Analysis of Crack Characteristics

#### 3.2.1. Crack Identification

**Figure 10:**
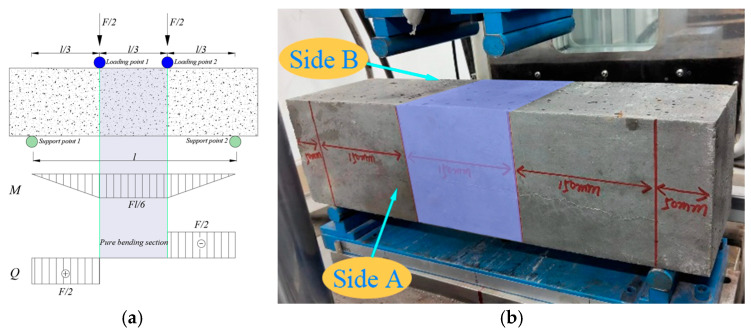
Bending section of the four-point fatigue test. (**a**) Internal force diagram, (**b**) test piece loading diagram.

The fatigue fracture of ceramsite concrete specimens under varying stress levels primarily occurred in the pure bending section (as shown in Figure 10).

**Figure 11:**
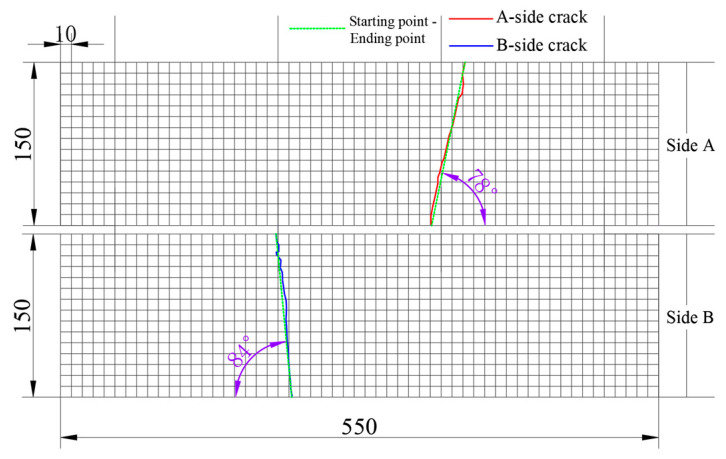
Crack diagram of specimen side A and B.

In order to enhance the study of the location and angle of cracks in the ceramsite concrete specimens following fracture, image acquisition technology was utilized to conduct a statistical examination and visualization of the cracks present on both surfaces of the specimens. The orientation of the cracks on Side A (550 mm × 150 mm) is indicated by red lines, while blue lines represent the crack orientation on Side B (550 mm × 150 mm). The figure illustrates the crack directions, with the angles of each crack clearly annotated, as demonstrated in Figure 11.

Each individual grid on the coordinate paper was 10 mm × 10 mm. The coordinate paper was cut to match the dimensions of the specimen, which were 550 mm in length, 150 mm in width, and 150 mm in height. The four edges of the coordinate paper were fully integrated and secured to the specimen. Additionally, due to the transparency of the coordinate paper, the cracks in the specimen could be accurately reproduced on the coordinate paper.

**Figure 12:**
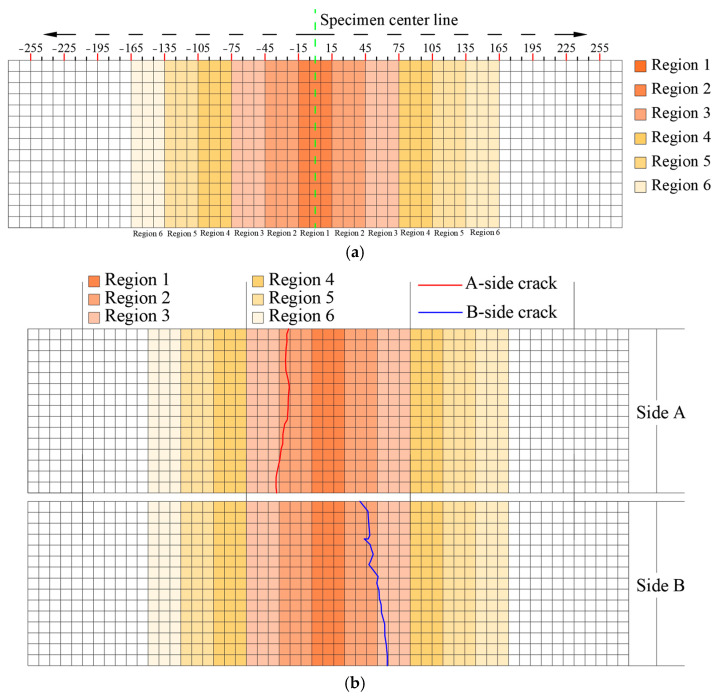
Regional division diagram. (**a**) Regional division, (**b**) crack diagram.

In order to facilitate the presentation and analysis of the precise locations of cracks, the side of the ceramic lightweight aggregate concrete specimen was divided into six regions. The specimen center functioned as the midpoint, with divisions occurring at intervals of 30 mm. The regions were defined as follows: Region 1 (−15, 15), Region 2 (−45, −15) and (15, 45), Region 3 (−75, −45) and (45, 75), Region 4 (−105, −75) and (75, 105), Region 5 (−135, −105) and (105, 135), and Region 6 (−165, −135) and (135, 165), as illustrated in Figure 12.

#### 3.2.2. Crack Location

(1) The influence of ceramsite size and content

**Figure 13:**
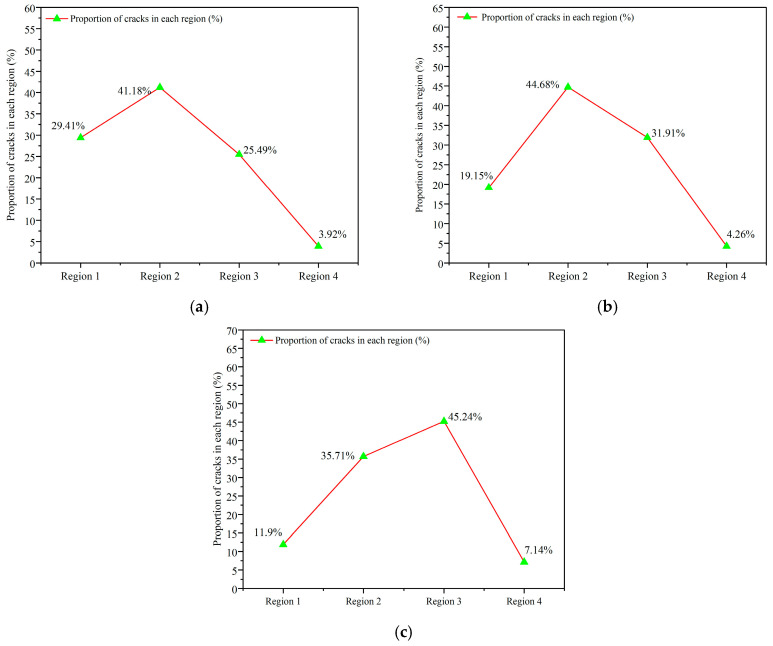
Statistics of crack locations with different ceramsite replacement rates. (**a**) Statistics of crack location with CRR3, (**b**) statistics of crack location with CRR2, (**c**) statistics of crack location with CRR1.

The location of cracks in concrete is fundamentally associated with its inhomogeneity, which may result from the spatial distribution of aggregates and variations in their properties. Significant differences exist between ceramsite and natural aggregates regarding their morphology, surface characteristics, and bonding properties with cement mortar. These differences significantly influence the paths by which cracks either circumvent or pass through ceramsite compared to natural aggregates. Furthermore, variations in the particle size and proportion of ceramsites can modify the internal composition of the concrete, potentially influencing the characteristics of cracks, including their locations. Therefore, it can be speculated that the crack location may be influenced by the ceramsite size and the ceramsite replacement rate. The regional division diagram illustrates the locations of cracks in the ceramsite concrete specimens subjected to fatigue tests at varying stress levels, as documented in Appendix A. The fatigue crack locations presented in Appendix A were categorized and subjected to statistical analysis based on the ceramsite replacement rate, as demonstrated in Figure 13.

Groups 1–4 correspond to CRR1, groups 5–8 correspond to CRR2, and groups 9–12 correspond to CRR3, as illustrated in Figure 13.

As depicted in Figure 13, the distribution of fatigue cracks across various regions under the CRR3 condition followed the proportion: region 2 > region 1 > region 3 > region 4. In the case of CRR2, the observed pattern was region 2 > region 3 > region 1 > region 4. For CRR1, the distribution was characterized by the following sequence: region 3 > region 2 > region 1 > region 4. These findings suggest that the occurrence of fatigue cracks in different regions of ceramsite concrete specimens, influenced by varying rates of ceramsite substitution, was predominantly concentrated in region 2, with region 3 also exhibiting a significant prevalence, followed by regions 1 and 4. Region 4, located in a more peripheral area of the specimen, demonstrated a comparatively low likelihood of crack formation, regardless of the ceramsite substitution rate. As a result, the incidence of cracks in region 4 remained unaffected and consistently with the lowest proportion. Conversely, the proportions of cracks in regions 1, 2, and 3 were influenced by the ceramsite substitution rate.

The analysis reveals that the prevalence of fatigue cracks was most pronounced in region 2 when utilizing ceramsites with replacement rates of 5–10 mm and 10–20 mm, with the only variation being the relative proportions of regions 1 and 3. An increase in the proportion of smaller ceramsites reduced the likelihood of larger aggregates obstructing crack formation, while simultaneously decreasing the transition zone at the concrete interface. Conversely, this increase enhanced the specific surface area of the ceramsites, thereby improving the adhesion between the ceramsites and the cement mortar. Ultimately, these factors facilitated a singular crack propagation process within the ceramsite concrete specimen. In contrast, the incorporation of 20–30 mm ceramsites produced a dual effect: it enhanced the transition zone at the concrete interface, while concurrently reducing the specific surface area of the ceramsites. This reduction in surface area resulted in diminished adhesion between the ceramsites and the cement mortar, thereby creating additional potential sites for the initiation of microcracks and pathways for crack propagation within the cement mortar. Moreover, the presence of larger ceramsites contributed to a more discrete crack propagation, which could hinder the progression of cracks to a certain extent, as they were compelled to navigate around the ceramsites and coarse aggregates. Consequently, the proportion of cracks in region 3 was correspondingly increased.

(2) The influence of stress level

**Figure 14:**
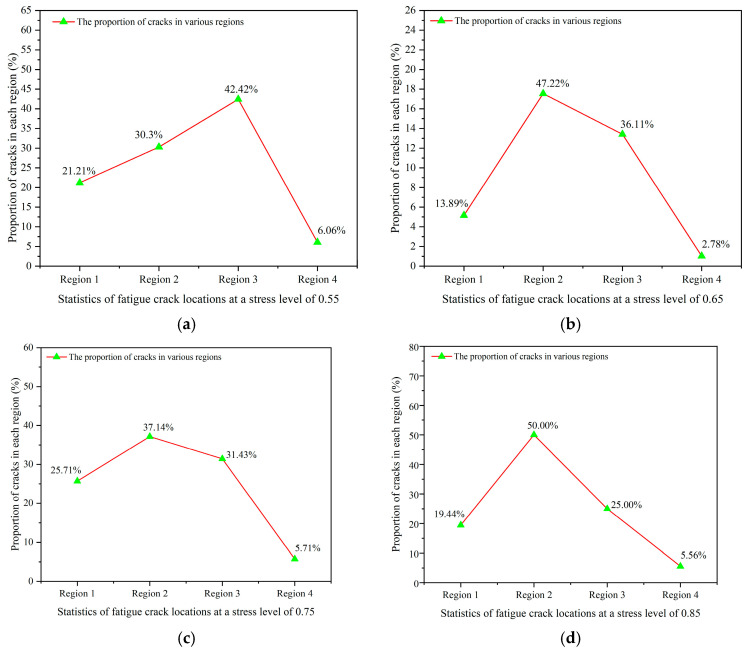
Statistics of crack locations under different stress levels. (**a**) The stress level was 0.55, (**b**) the stress level was 0.65, (**c**) the stress level was 0.75, (**d**) the stress level was 0.85.

This study presents a statistical analysis of the primary concentration of fatigue cracks in the ceramsite concrete specimens, derived from crack locations identified in 12 sets of tests conducted at varying stress levels, as depicted in Figure 14.

As illustrated in Figure 14, the distribution of fatigue cracks across the different regions at a stress level of 0.55 exhibited the following hierarchy: region 3 > region 2 > region 1 > region 4. Upon increasing the stress level to 0.65, the order of crack concentration shifted to region 2 > region 3 > region 1 > region 4. At a stress level of 0.75, the predominant concentration of fatigue cracks remained consistent with the previous findings, retaining the order region 2 > region 3 > region 1 > region 4. This pattern continued at a stress level of 0.85, where the order of concentration persisted as region 2 > region 3 > region 1 > region 4. Furthermore, it is noteworthy that at stress levels of 0.55 and 0.66, the fatigue cracks did not manifest during the initial fatigue cycles; rather, they emerged only after a certain level of fatigue cycle accumulation had been reached.

It can be deduced that the distribution of fatigue crack locations in the ceramsite concrete specimens subjected to varying stress levels approximately followed the following hierarchy: region 2 ≈ region 3 > region 1 > region 4. This distribution is consistent with the observed patterns of fatigue crack locations across the different ceramsite replacement rates. It is apparent that the regions in which fatigue cracks manifested were relatively unaffected by variations in stress levels.

#### 3.2.3. Crack Angle

(1) The influence of ceramsite size and content

**Figure 15:**
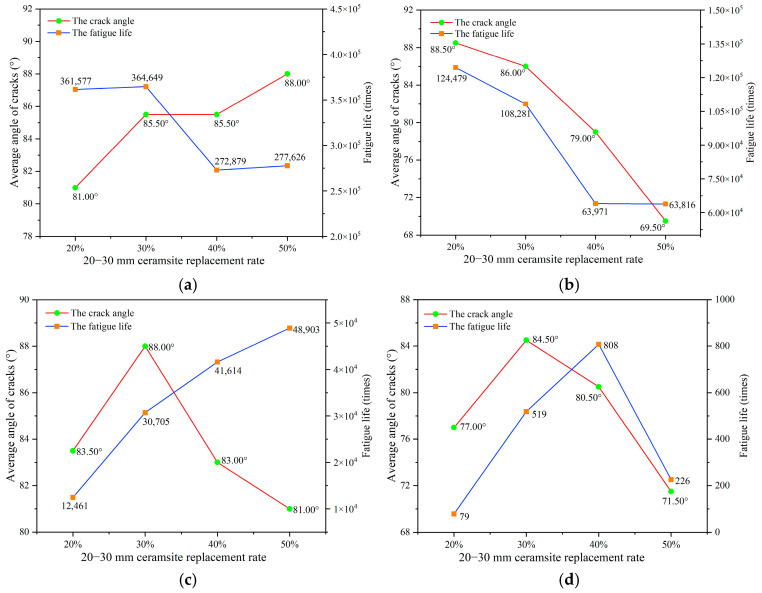
The relationship between fatigue life and the average crack angle in relation to CRR1. (**a**) The stress level was 0.55, (**b**) the stress level was 0.65, (**c**) the stress level was 0.75, (**d**) the stress level was 0.85.

**Figure 16:**
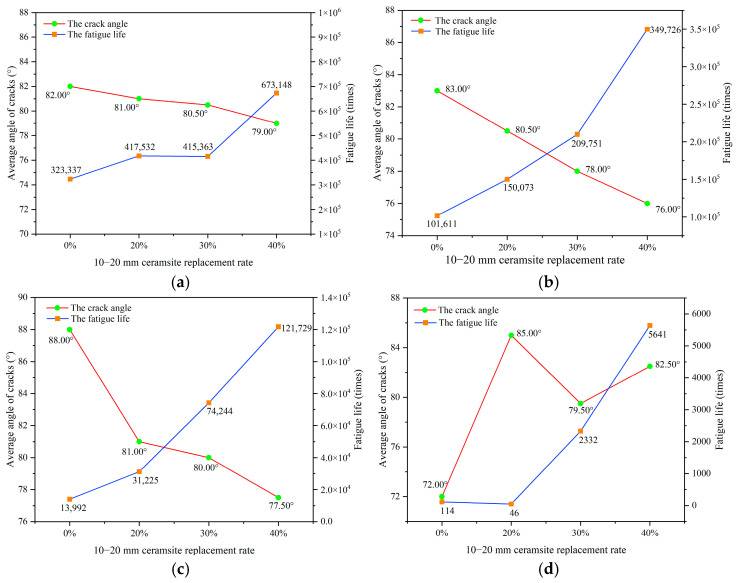
The relationship between fatigue life and the average crack angle in relation to CRR2. (**a**) The stress level was 0.55, (**b**) the stress level was 0.65, (**c**) the stress level was 0.75, (**d**) the stress level was 0.85.

**Figure 17:**
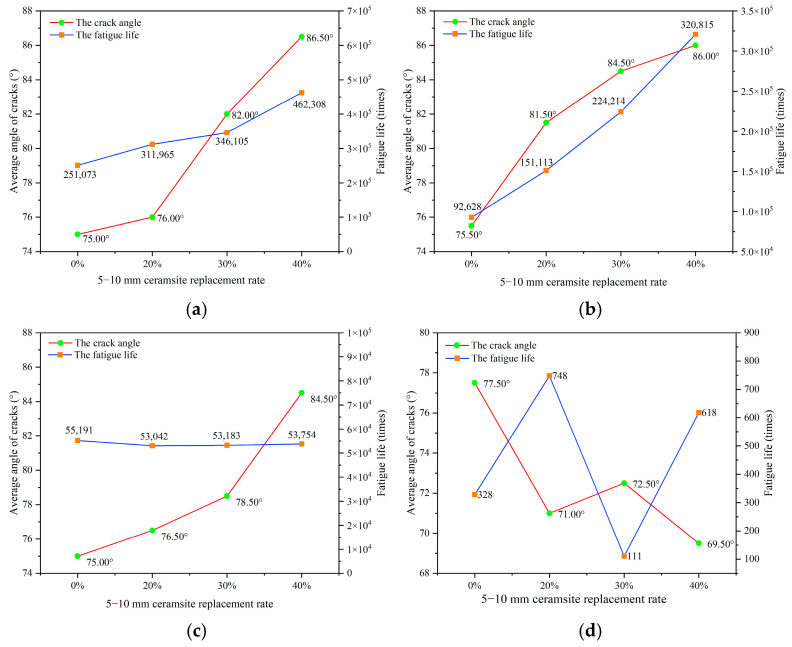
The relationship between fatigue life and the average crack angle in relation to CRR3. (**a**) The stress level was 0.55, (**b**) the stress level was 0.65, (**c**) the stress level was 0.75, (**d**) the stress level was 0.85.

The objective of this research was to investigate the influence of ceramsite properties, particularly particle size and content, on the angle of fatigue cracks in concrete. To facilitate this analysis, statistical data concerning crack angles from twelve experimental sets conducted under different stress levels were gathered, as illustrated in Figure 11 and detailed in Appendix B. The fatigue life and crack angle as the replacement rate of ceramsite with various sizes was increased are illustrated in Figure 15, Figure 16 and Figure 17.

A notable relationship existed between the crack angles and fatigue life for CCR2 and CRR3. However, this relationship tended to weaken as the stress level rose, especially at a stress level of 0.85. This phenomenon occurred because the breakage of ceramsite and aggregate began to take place at higher stress levels, leading to increased uncertainty in crack propagation.

**Figure 18:**
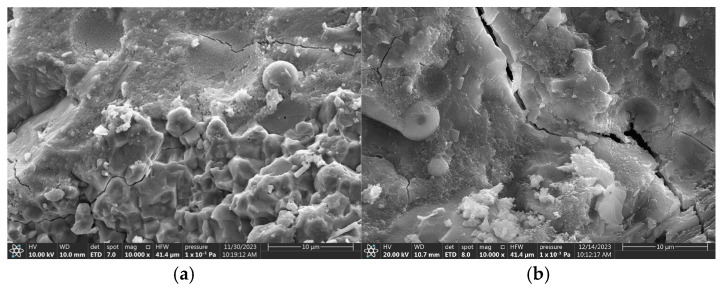
SEM images (natural aggregate and 5–10 mm ceramsite). (**a**) At low to moderate stress levels (0.55, 0.65, 0.75), (**b**) at a higher stress level (0.85).

**Figure 19:**
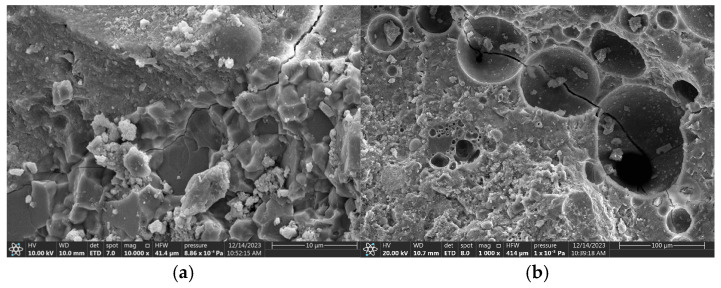
SEM images (natural aggregate and 10–20 mm ceramsite). (**a**) At low to moderate stress levels (0.55, 0.65, 0.75), (**b**) at a higher stress level (0.85).

**Figure 20:**
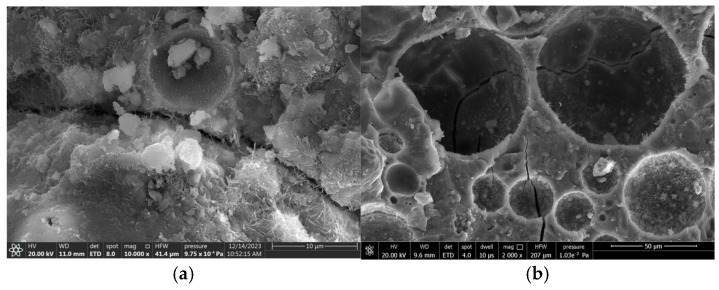
SEM images (natural aggregate and 20–30 mm ceramsite). (**a**) At low to moderate stress levels (0.55, 0.65, 0.75), (**b**) at a higher stress level (0.85).

Figure 18, Figure 19 and Figure 20 provide SEM images of the failure surface of the specimen at different stress levels. It can be observed that cracks did not propagate through the ceramsite under conditions of medium and low stress levels. Moreover, at low and moderate stress levels, the reduction in particle size correlated with the increasing distance of crack formation from the particle itself. This phenomenon can be attributed to the fact that the smaller ceramsite possessed a reduced surface area, which enhanced their bonding with the interface transition zone of the cement mortar. Consequently, this improved connectivity diminished the likelihood of crack development within the interface transition zone.

Ceramsites measuring 5–10 mm demonstrated the highest specific surface area in comparison to the other ceramsite sizes. The enhancement of these ceramsites within the concrete transition zone was positively correlated with the increase in the CRR3 value, which in turn contributed to an extended fatigue life. Nevertheless, due to their relatively small dimensions, the influence of the 5–10 mm ceramsites on crack bending was minimal. As the CRR3 value escalated, the distribution of ceramsites throughout the concrete matrix became increasingly uniform, resulting in a propensity for crack propagation to occur in a vertical direction. This relationship is further illustrated in Figure 17, where a positive correlation between crack angle and fatigue life is evident.

As the dimensions of the ceramsite increased, particularly in the range of 10–20 mm, the probability of cracks circumventing these ceramsites during their propagation also escalated. This occurrence led to the formation of curved crack trajectories and a decrease in the crack angle, which in turn extended the length of the crack path and prolonged the failure process, thereby improving fatigue life. This effect was further amplified with an increase in the CRR2 value. Therefore, as depicted in Figure 16, a negative correlation was evident between the crack angle and fatigue life.

As the size of ceramsite continues to increased (particularly for 20–30 mm ceramsites), the larger size ceramsites may have influenced the directionality of the crack path when cracks approached them. This phenomenon amplified the uncertainty in the propagation process. Furthermore, given identical volumetric conditions, the particle number of CRR1 ceramsites was comparatively lower, resulting in a distribution of CRR1 ceramsites that exhibited a greater degree of randomness. This randomness further amplified the uncertainty regarding the influence of CRR1 on fatigue life. Consequently, these two factors led to a lack of correlation between crack angle and fatigue life.

The absolute value of the Pearson coefficient was utilized to compare the relationship between crack angle and fatigue life.

Table 13 shows that the absolute values of the Pearson coefficients at stress levels of 0.55, 0.65, and 0.75 all exceeded 0.65, signifying a robust correlation between crack angle and fatigue life at these specific stress levels. Conversely, the absolute value of the Pearson coefficient at the stress level of 0.85 was only 0.34; therefore, the corresponding plots for this stress level are omitted from the manuscript.

**Figure 21:**
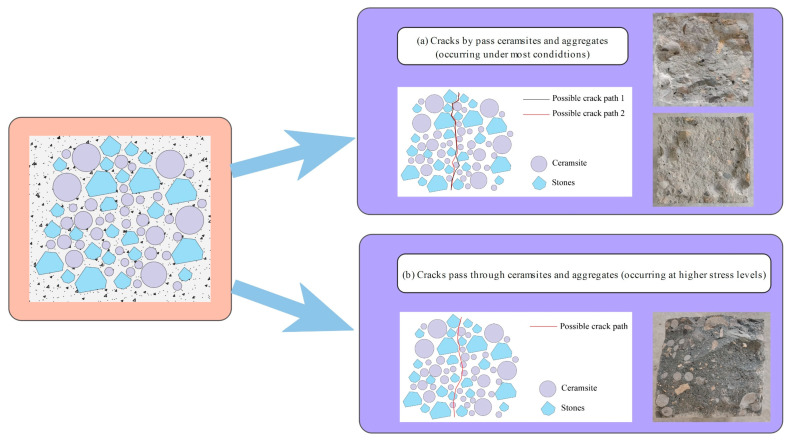
The CRR3 crack simulation path.

The influence of ceramic particle content, as illustrated in Figure 17, specifically within the range of 5–10 mm, indicates that an increase in the concentration of these particles correlated with an increase in the crack angle at stress levels of 0.55, 0.65, and 0.75. Additionally, the orientation of the crack transitioned from bending expansion to vertical expansion. This behavior can be explained by the improved uniformity in the distribution of ceramsites within the concrete specimen as their concentration increased. During the process of crack propagation, cracks tend to follow paths of lower strength. The presence of aggregates along a crack’s path can lead to a deflection in the crack’s direction. As a result, a decreased likelihood of encountering larger aggregate particles during propagation reduces the potential for directional changes in the crack. Moreover, the uniformly distributed ceramsites demonstrated strong adhesion to the cement mortar. When subjected to vertical loads, the energy required for crack propagation in the vertical direction was minimized, thereby promoting a more linear crack trajectory. Furthermore, the time required for the crack to traverse the cross-section from initiation to completion was diminished, suggesting a shorter duration spent in identifying the path that incurred the least energy loss, as illustrated in Figure 21.

**Figure 22:**
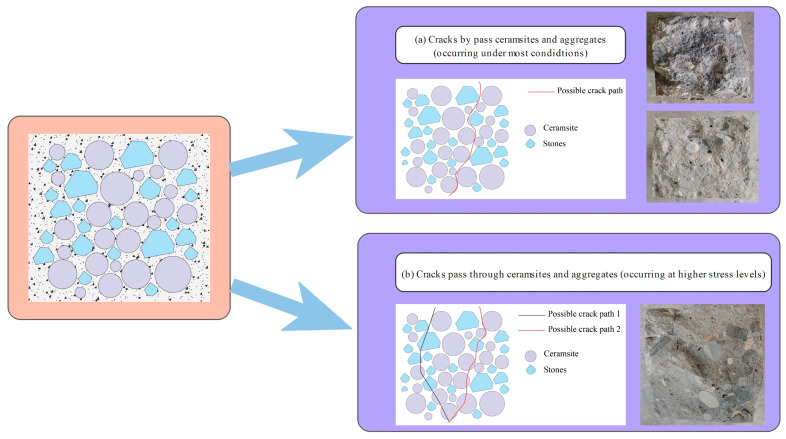
The CRR2 crack simulation path.

The influence of the 10–20 mm ceramsite content is clearly depicted in Figure 16, which demonstrates that at stress levels of 0.55, 0.65, and 0.75, an increase in the proportion of 10–20 mm ceramic particles was associated with a decrease in the crack angle and a more pronounced curvature of the crack trajectory. This phenomenon can be attributed to the fact that a higher content of 10–20 mm ceramsites resulted in an increased presence of medium-sized aggregates within the ceramsite concrete specimen. As cracks propagate, they are more likely to encounter larger aggregates, which influences their trajectory. The crack path will randomly seek the route that minimizes energy loss, resulting in a more curved crack path, as illustrated in Figure 22.

**Figure 23:**
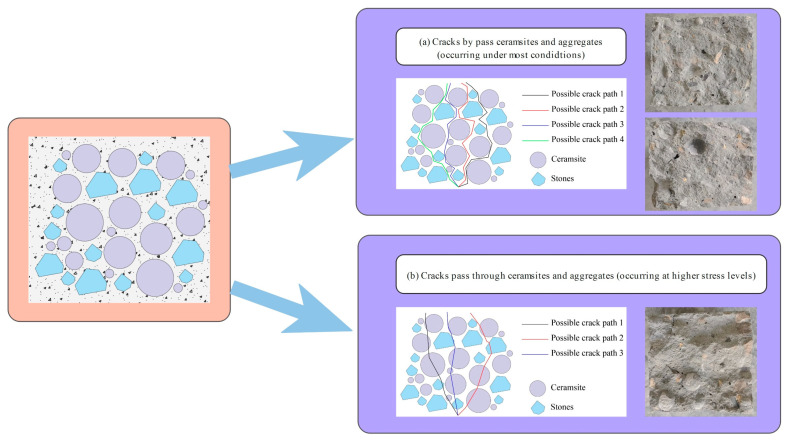
The CRR1 crack simulation path.

As illustrated in Figure 15, the influence of ceramsite content within the range of 20–30 mm indicates that an increase in ceramsite concentration led to variability in the angles of the resultant cracks, without a clear pattern emerging. This occurrence can be explained by the presence of larger ceramsites, which resulted in an uneven distribution and, subsequently, inconsistencies in the mortar distribution among the aggregates in the ceramsite concrete specimens. Consequently, this may have given rise to multiple pathways for crack propagation, as shown in Figure 23.

The results demonstrate a notable correlation between crack angles and the size and content of ceramsite. Analyzing the crack angle enhances the comprehension of fatigue crack propagation in ceramsite lightweight concrete.

(2) The influence of stress level

**Figure 24:**
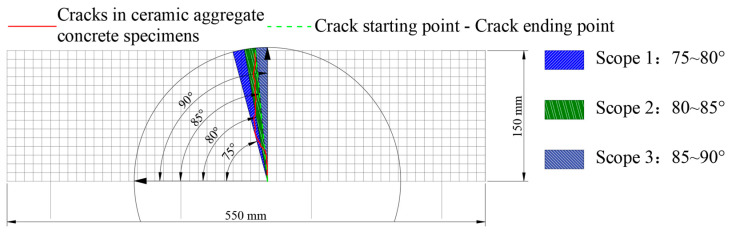
Schematic diagram of angle range division of fatigue cracks.

In order to elucidate the impact of stress levels on crack angles, the cracks identified in lightweight aggregate concrete fatigue specimens were classified into three specific ranges of crack angles, as illustrated in Figure 24. These ranges were delineated as follows: Angle Range 1 (75° to 80°), Angle Range 2 (80° to 85°), and Angle Range 3 (85° to 90°).

**Figure 25:**
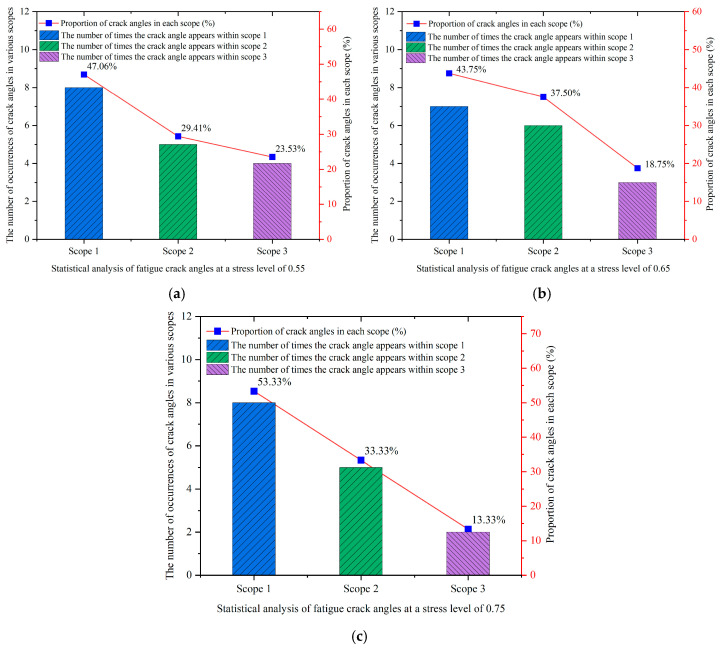
Statistics of fatigue crack angles under different stress levels. (**a**) The stress level was 0.55, (**b**) the stress level was 0.65, (**c**) the stress level was 0.75.

The frequency and proportion of fatigue cracks that manifested at various angles across the different stress levels were determined, as illustrated in Figure 25.

Figure 25 demonstrates that the distribution of fatigue crack angles across the different stress levels exhibited a consistent pattern. Specifically, the frequency of crack angles, ranked from highest to lowest occurrence, was categorized into three ranges: range 1 (75–80°), range 2 (80–85°), and range 3 (85–90°). Additionally, it is observed that with an increase in stress levels, there was a significant reduction in both the frequency and proportion of fatigue crack angles within range 3. This observation aligns with the analysis presented in Section 3.2.2 regarding the impact of stress levels on the location of fatigue cracks. It is well known that cracks typically occur near the mid-span of a specimen, due to the maximum bending moment experienced in that region under bending fatigue loading. However, as stress levels rise, the phenomenon of stress concentration at the support points on either side of the specimen becomes increasingly pronounced. Considering that concrete is a heterogeneous material, it is possible for weak zones to form in proximity to the support points. The interplay of stress concentration and the accumulation of damage within these weak zones heightens the risk of initiating and propagating fatigue cracks in the areas surrounding the support points during fatigue testing, thus increasing the probability of crack development in these regions. Therefore, it can be concluded that the impact of stress levels on fatigue cracks in lightweight aggregate concrete is predominantly reflected in the curvature of the crack trajectory. As stress levels continue to escalate, the load applied to the concrete specimen correspondingly increases, resulting in heightened internal forces within the specimen. The internal micro-cracks surrounding the aggregates in the concrete do not have adequate time to propagate at elevated stress levels [18]. Consequently, the increased internal forces exerted on the aggregates—comprising both ceramsites and natural aggregates—elevate the likelihood of aggregate cracking during fatigue testing. As a result, fatigue cracks at higher stress levels not only circumvent the aggregates but also traverse through them transversely. This occurrence contribute to an increase in the randomness of crack paths as stress levels rise, thereby complicating the trajectories of fatigue cracks. However, it is noteworthy, as illustrated in Figure 16, that with the progressive increase in stress levels, the angular variation in cracks became more pronounced during the transition of the ceramsite replacement rate from 0 to 40% within the 10–20 mm range. Thus, it can be concluded that stress levels also exert influence on the angular dimensions of fatigue cracks in ceramsite lightweight aggregate concrete.

## 4. Conclusions

This research examined the influence of ceramsite size, ceramsite content, and stress levels on the propagation of cracks—particularly regarding their location and angle—in ceramsite lightweight concrete subjected to fatigue conditions. The study yielded the following conclusions:

- The impact of varying replacement rates of 5–10 mm ceramsite on the occurrence of fatigue cracks was examined, revealing a significant increase in crack incidence within the region 15 mm to 45 mm from the sample’s centerline, which reached 41.2%. This rise in the proportion of smaller particles was associated with an increase in the angle of the fatigue cracks, resulting in a shift in the direction of crack propagation from a bending orientation to a more vertical orientation.
- The impact of varying replacement rates of ceramsite measuring 10–20 mm on the location of fatigue cracks demonstrated a notable consistency with the findings observed at the replacement rate of 5–10 mm. Notably, the highest proportion recorded was 44.7%. Nevertheless, an increase in the proportion of medium-sized aggregates influenced the trajectory of the cracks, leading to a reduction in the crack angle and a tendency for the crack path to exhibit a more curved configuration.
- The investigation into the impact of different replacement rates of 20–30 mm ceramsites indicated that the locations and angles of fatigue cracks exhibited variability throughout their propagation. The introduction of larger aggregates seemed to hinder the vertical progression of cracks, resulting in a higher average number of cracks within the area ranging from 45 mm to 75 mm from the centerline of the specimen. Additionally, the use of larger aggregate sizes intensified the curvature of the crack propagation path. In general, the effect of ceramsite replacement rate on crack location was less significant than that of ceramsite size.
- The spatial distribution of fatigue crack locations across different regions subjected to varying stress levels corresponded with the distribution observed in regions with differing rates of ceramsite substitution. This correlation indicates that the initiation of fatigue crack positions was largely independent of the applied stress levels. Nevertheless, an elevation in stress levels led to a more complex and curved trajectory of the fatigue cracks. Therefore, the influence of stress levels on fatigue cracks was predominantly reflected in the extent of curvature of their paths.

Future research will employ numerical simulation techniques to determine the spatial distribution of ceramsite and natural aggregates, thereby facilitating more comprehensive and in-depth investigations into the effects of ceramsite on the location and angle of fatigue cracks.

## Figures and Tables

**Table 1:** Physical properties of cement.

Fineness (%)	Standard Viscosity (%)	Stability	Setting Time (min)		Flexural Strength (MPa)	Compressive Strength (MPa)
		Cake Testing Method	Initial	Final	3-Day	3-Day
5.9	28.3	Qualified	215	275	5.7	26.8

**Table 2:** Technical properties of aggregates.

Crushing Value (%)	Wear Loss (%)	Robustness (%)	Needle Like Particle Content (%)	Water Absorption Rate (%)
14.4	16.2	6.2	6.1	0.81

**Table 3:** Technical properties of ceramsites.

Apparent Density (kg/m^3^)	Cylinder Compression Strength (MPa)	Water Absorption Rate (%)	Mud Content (%)	Cl^−^ (%)
1950	16.4	9.8	1.0	0.01

**Table 4:** Technical properties of silica fume.

Density (kg/m^3^)	Activity Index (%)	Specific Surface Area (m^2^/g)	Water Demand Ratio (%)	Alkali Content (%)	SiO_2_ Content (%)	SO_3_ Content (%)	Cl^−^ (%)
2200	108	20,000	123	0.23	92	0.33	0.005

**Table 5:** Physical properties of fly ash.

Density (g/m^3^)	Bulk Density (g/m^3^)	Specific Surface Area (m^2^/g)	Water absorption Capacity (%)	Water Demand	Water Content
2.1	0.79	3500	106	≤100%	≤0.9%

**Table 6:** Technical properties of river sand.

Particle Size (mm)	Fineness Modulus	Bulk Density (kg/m^3^)	Apparent Density (kg/m^3^)
<5	2.7	1392	2588

**Table 7:** Technical properties of water-reducing agent.

Technical Indicators		Detection Result
Water-reducing rate (%)		35
Bleeding rate (%)		29
Gas content (%)		4.0
Difference in condensation time (min)	Initial setting	+20
	Final setting	−30
Compressive strength ratio (%)	1 d	220
	3 d	183
	7 d	180
	28 d	165

**Table 8:** Different aggregate contents.

No.	CRR1	CRR2	CRR3
N-1	20%	10%	10%
N-2	30%	10%	10%
N-3	40%	10%	10%
N-4	50%	10%	10%
N-5	30%	0%	10%
N-6	30%	20%	10%
N-7	30%	30%	10%
N-8	30%	40%	10%
N-9	30%	10%	0%
N-10	30%	10%	20%
N-11	30%	10%	30%
N-12	30%	10%	40%

**Table 9:** Parameters of the fatigue tests.

Loading Frequency (Hz)	Stress Level (MPa)	Stress Ratio	Stress Loading Speed (mm/s)
10	0.55, 0.65, 0.75, 0.85	0.1	0.005

**Table 10:** Analysis of variance (ANOVA) of the fatigue life of CRR1.

Stress Level (MPa)	SS		d_f_	MS	F	*p*-Value
0.55	SSB	3.9 × 10^10^	3	1.3 × 10^10^	4.63	0.016
	SSW	4.5 × 10^10^	16	2.8 × 10^9^		
0.65	SSB	1.4 × 10^10^	3	4.8 × 10^9^	15.48	<0.001
	SSW	5.0 × 10^9^	16	3.1 × 10^8^		
0.75	SSB	3.8 × 10^9^	3	1.3 × 10^9^	62.46	<0.001
	SSW	3.2 × 10^8^	16	2.0 × 10^7^		
0.85	SSB	1.6 × 10^6^	3	5.2 × 10^5^	5.35	0.010
	SSW	1.6 × 10^6^	16	9.8 × 10^4^		

**Table 11:** Analysis of variance (ANOVA) of the fatigue life of CRR2.

Stress Level (MPa)	SS		d_f_	MS	F	*p*-Value
0.55	SSB	3.8 × 10^11^	4	9.4 × 10^10^	14.11	<0.001
	SSW	1.3 × 10^11^	20	6.7 × 10^9^		
0.65	SSB	2.1 × 10^11^	4	5.2 × 10^10^	52.07	<0.001
	SSW	2.0 × 10^10^	20	1.0 × 10^9^		
0.75	SSB	3.8 × 10^10^	4	9.6 × 10^9^	43.62	<0.001
	SSW	4.4 × 10^9^	20	2.2 × 10^8^		
0.85	SSB	1.1 × 10^8^	4	2.8 × 10^7^	14.25	<0.001
	SSW	4.0 × 10^7^	20	2.0 × 10^6^		

**Table 12:** Analysis of variance (ANOVA) of the fatigue life of CRR3.

Stress Level (MPa)	SS		d_f_	MS	F	*p*-Value
0.55	SSB	1.2 × 10^11^	4	3.0 × 10^10^	12.69	<0.001
	SSW	4.7 × 10^10^	20	2.4 × 10^9^		
0.65	SSB	1.8 × 10^11^	4	4.4 × 10^10^	59.55	<0.001
	SSW	1.5 × 10^10^	20	7.4 × 10^8^		
0.75	SSB	2.1 × 10^9^	4	5.4 × 10^8^	2.91	0.047
	SSW	3.7 × 10^9^	20	1.8 × 10^8^		
0.85	SSB	1.3 × 10^6^	4	3.1 × 10^5^	2.89	0.048
	SSW	2.2 × 10^6^	20	1.1 × 10^5^		

**Table 13:** The absolute value of the Pearson coefficient between crack angle and fatigue life.

**Stress Level**	0.55	0.65	0.75	0.85
**Absolute Value of Pearson Coefficient**	0.86	0.92	0.66	0.34

**Figure A1:**
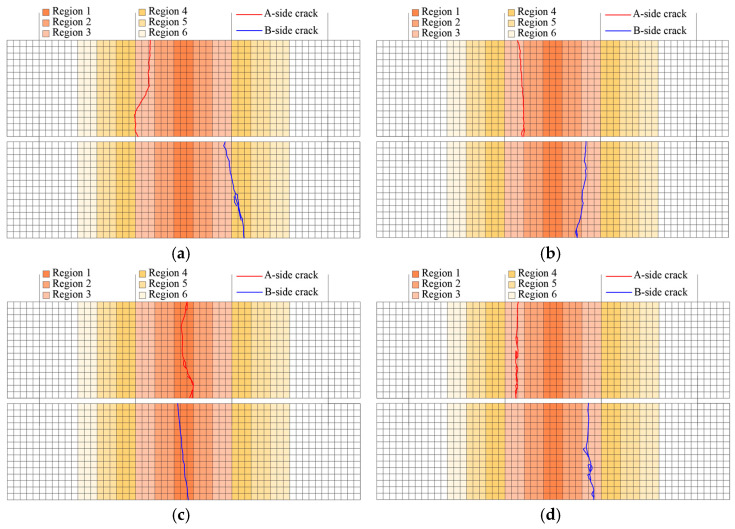
The positions of cracks in groups 1–4 at a stress level of 0.55. (**a**) C1 = 20%; C2 = 10%; C3 = 10%, (**b**) C1 = 30%; C2 = 10%; C3 = 10%, (**c**) C1 = 40%; C2 = 10%; C3 = 10%, (**d**) C1 = 50%; C2 = 10%; C3 = 10%.

**Figure A2:**
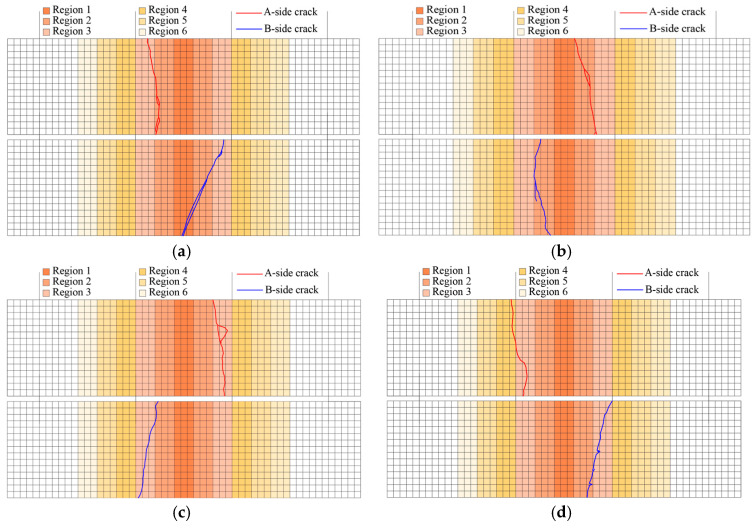
The positions of cracks in groups 5–8 at a stress level of 0.55. (**a**) C1 = 30%; C2 = 0%; C3 = 10%, (**b**) C1 = 30%; C2 = 20%; C3 = 10%, (**c**) C1 = 30%; C2 = 30%; C3 = 10%, (**d**) C1 = 30%; C2 = 40%; C3 = 10%.

**Figure A3:**
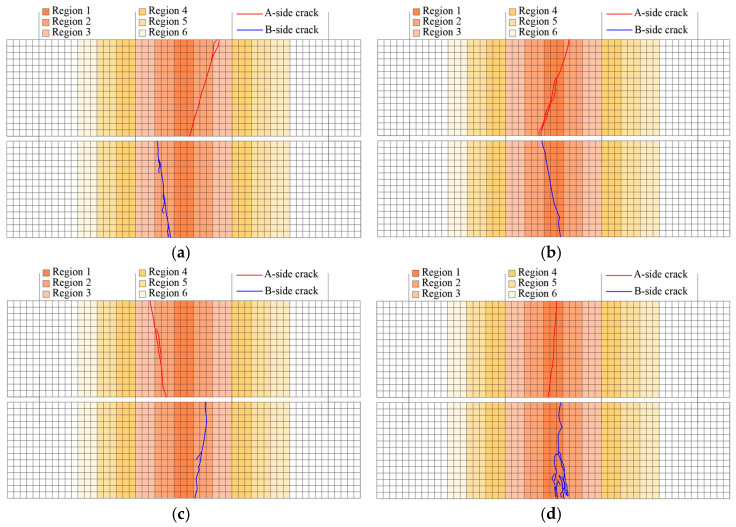
The positions of cracks in groups 9–12 at a stress level of 0.55. (**a**) C1 = 30%; C2 = 10%; C3 = 0%, (**b**) C1 = 30%; C2 = 10%; C3 = 20%, (**c**) C1 = 30%; C2 = 10%; C3 = 30%, (**d**) C1 = 30%; C2 = 10%; C3 = 40%.

**Figure A4:**
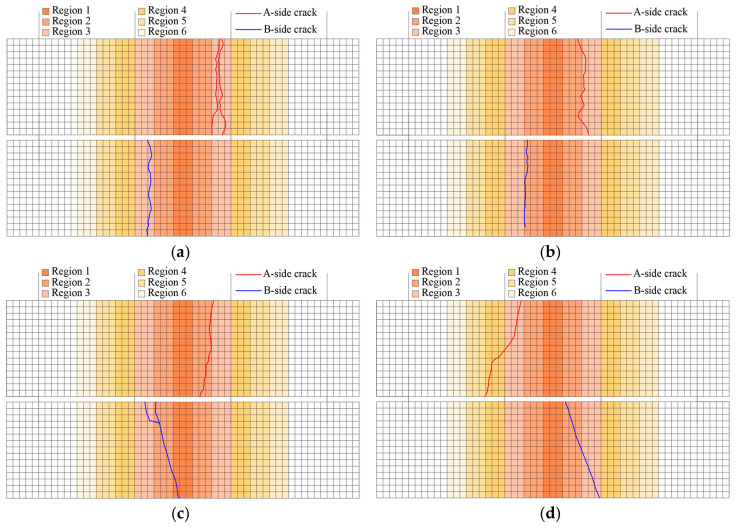
The positions of cracks in groups 1–4 at a stress level of 0.65. (**a**) C1 = 20%; C2 = 10%; C3 = 10%, (**b**) C1 = 30%; C2 = 10%; C3 = 10%, (**c**) C1 = 40%; C2 = 10%; C3 = 10%, (**d**) C1 = 50%; C2 = 10%; C3 = 10%.

**Figure A5:**
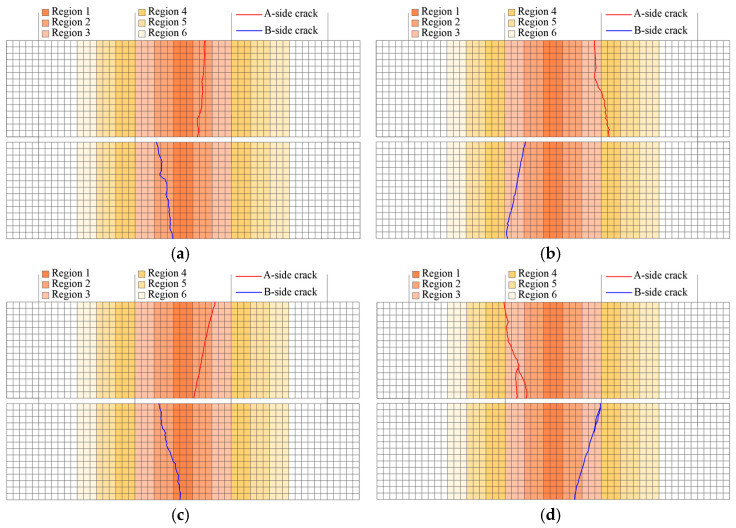
The positions of cracks in groups 5–8 at a stress level of 0.65. (**a**) C1 = 30%; C2 = 0%; C3 = 10%, (**b**) C1 = 30%; C2 = 20%; C3 = 10%, (**c**) C1 = 30%; C2 = 30%; C3 = 10%, (**d**) C1 = 30%; C2 = 40%; C3 = 10%.

**Figure A6:**
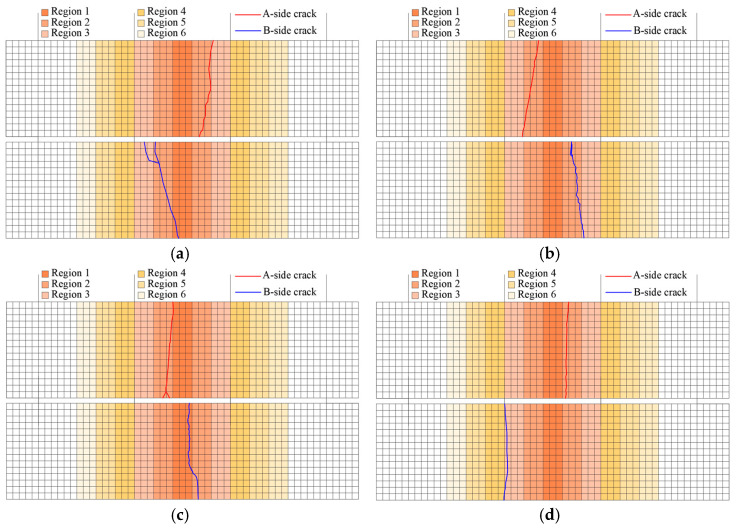
The positions of cracks in groups 9–12 at a stress level of 0.65. (**a**) C1 = 30%; C2 = 10%; C3 = 0%, (**b**) C1 = 30%; C2 = 10%; C3 = 20%, (**c**) C1 = 30%; C2 = 10%; C3 = 30%, (**d**) C1 = 30%; C2 = 10%; C3 = 40%.

**Figure A7:**
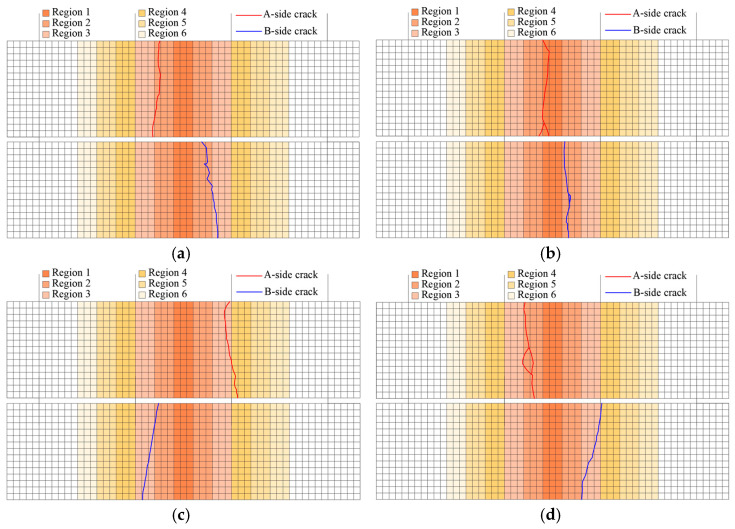
The positions of cracks in groups 1–4 at a stress level of 0.75. (**a**) C1 = 20%; C2 = 10%; C3 = 10%, (**b**) C1 = 30%; C2 = 10%; C3 = 10%, (**c**) C1 = 40%; C2 = 10%; C3 = 10%, (**d**) C1 = 50%; C2 = 10%; C3 = 10%.

**Figure A8:**
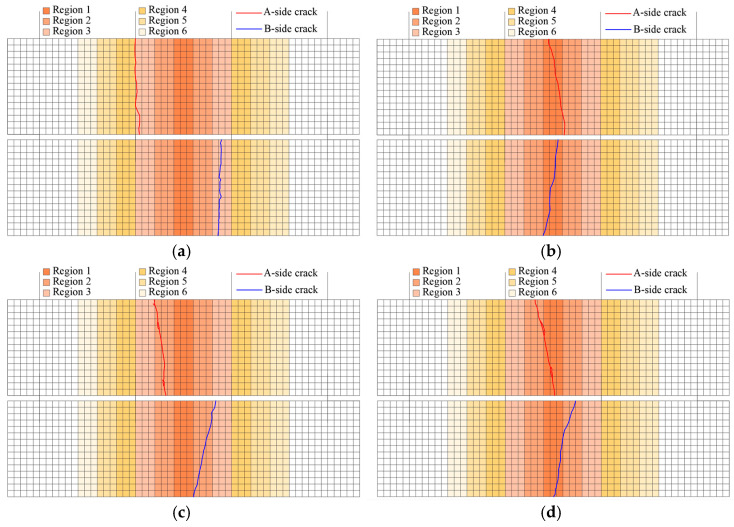
The positions of cracks in groups 5–8 at a stress level of 0.75. (**a**) C1 = 30%; C2 = 0%; C3 = 10%, (**b**) C1 = 30%; C2 = 20%; C3 = 10%, (**c**) C1 = 30%; C2 = 30%; C3 = 10%, (**d**) C1 = 30%; C2 = 40%; C3 = 10%.

**Figure A9:**
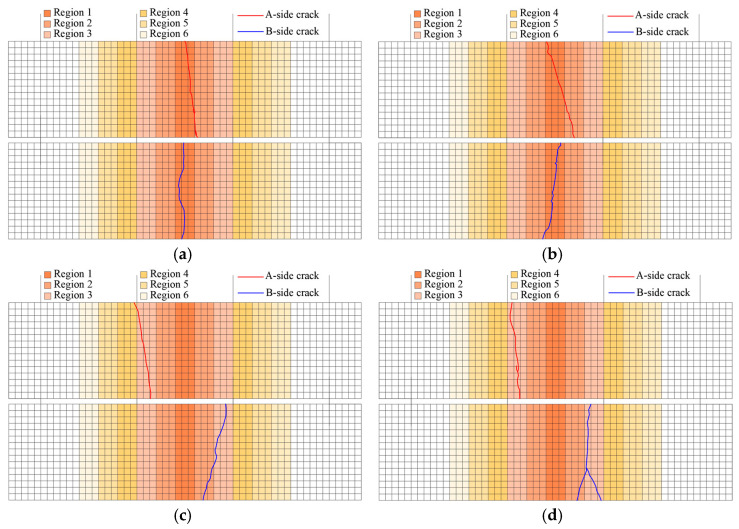
The positions of cracks in groups 9–12 at a stress level of 0.75. (**a**) C1 = 30%; C2 = 10%; C3 = 0%, (**b**) C1 = 30%; C2 = 10%; C3 = 20%, (**c**) C1 = 30%; C2 = 10%; C3 = 30%, (**d**) C1 = 30%; C2 = 10%; C3 = 40%.

**Figure A10:**
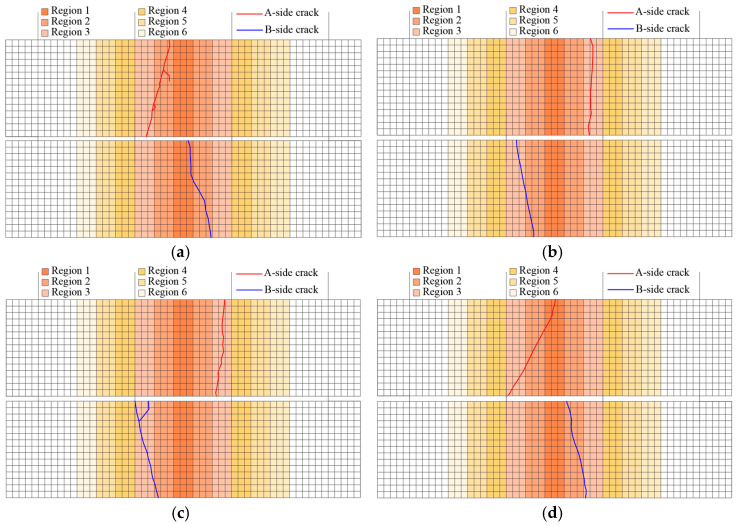
The positions of cracks in groups 1–4 at a stress level of 0.85. (**a**) C1 = 20%; C2 = 10%; C3 = 10%, (**b**) C1 = 30%; C2 = 10%; C3 = 10%, (**c**) C1 = 40%; C2 = 10%; C3 = 10%, (**d**) C1 = 50%; C2 = 10%; C3 = 10%.

**Figure A11:**
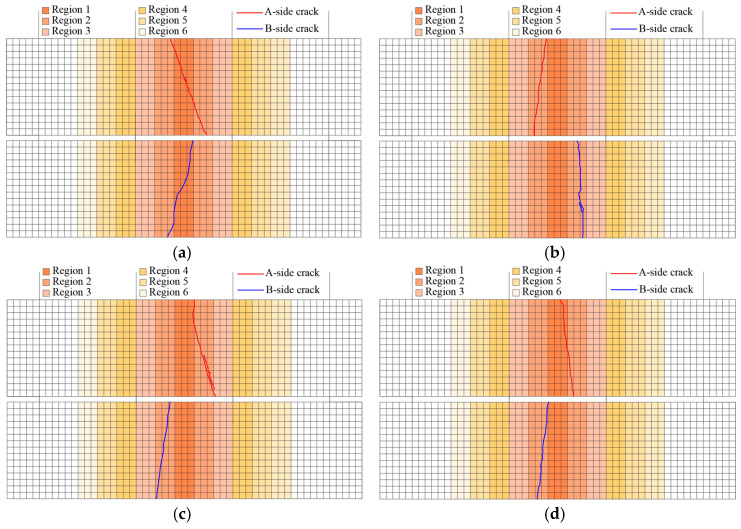
The positions of cracks in groups 5–8 at a stress level of 0.85. (**a**) C1 = 30%; C2 = 0%; C3 = 10%, (**b**) C1 = 30%; C2 = 20%; C3 = 10%, (**c**) C1 = 30%; C2 = 30%; C3 = 10%, (**d**) C1 = 30%; C2 = 40%; C3 = 10%.

**Figure A12:**
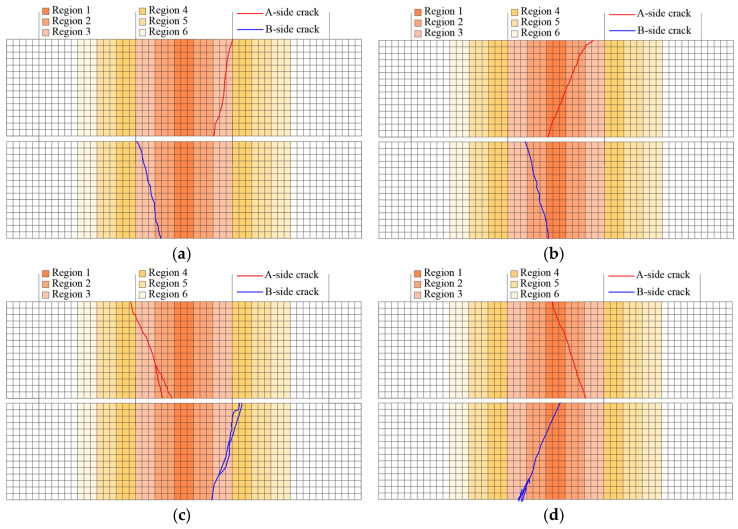
The positions of cracks in groups 9–12 at a stress level of 0.85. (**a**) C1 = 30%; C2 = 10%; C3 = 0%, (**b**) C1 = 30%; C2 = 10%; C3 = 20%, (**c**) C1 = 30%; C2 = 10%; C3 = 30%, (**d**) C1 = 30%; C2 = 10%; C3 = 40%.

**Figure A13:**
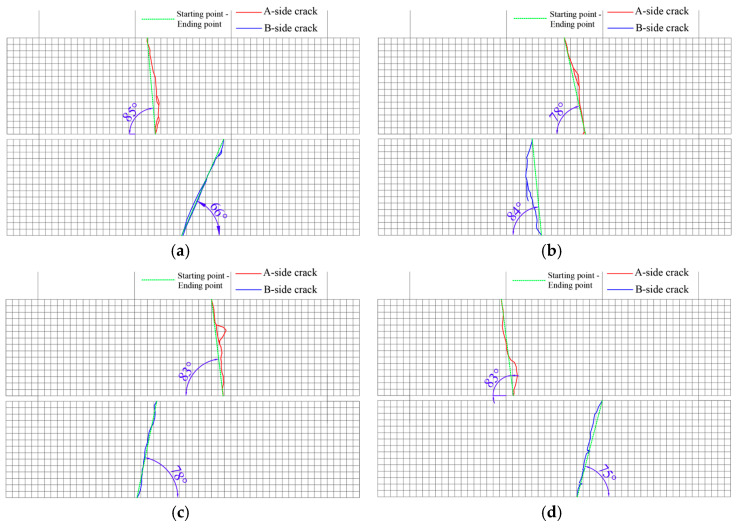
Crack angles of groups 5–8 at a stress level of 0.55. (**a**) C1 = 30%; C2 = 0%; C3 = 10%, (**b**) C1 = 30%; C2 = 20%; C3 = 10%, (**c**) C1 = 30%; C2 = 30%; C3 = 10%, (**d**) C1 = 30%; C2 = 40%; C3 = 10%.

**Figure A14:**
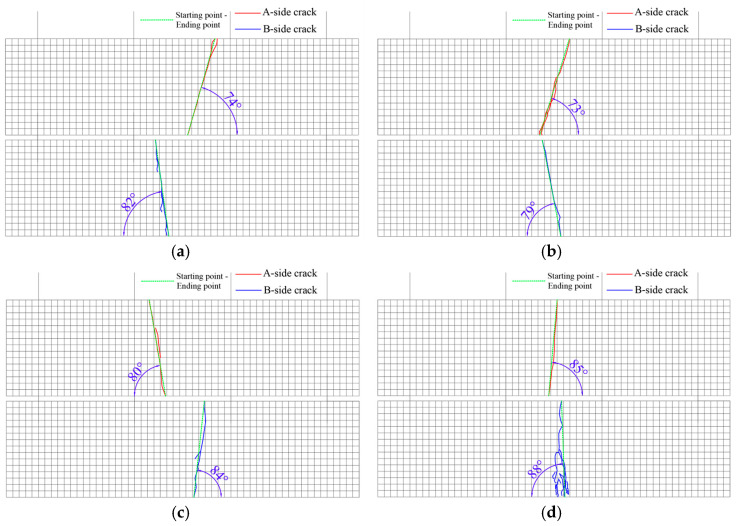
Crack angles of groups 9–12 at a stress level of 0.55. (**a**) C1 = 30%; C2 = 10%; C3 = 0%, (**b**) C1 = 30%; C2 = 10%; C3 = 20%, (**c**) C1 = 30%; C2 = 10%; C3 = 30%, (**d**) C1 = 30%; C2 = 10%; C3 = 40%.

**Figure A15:**
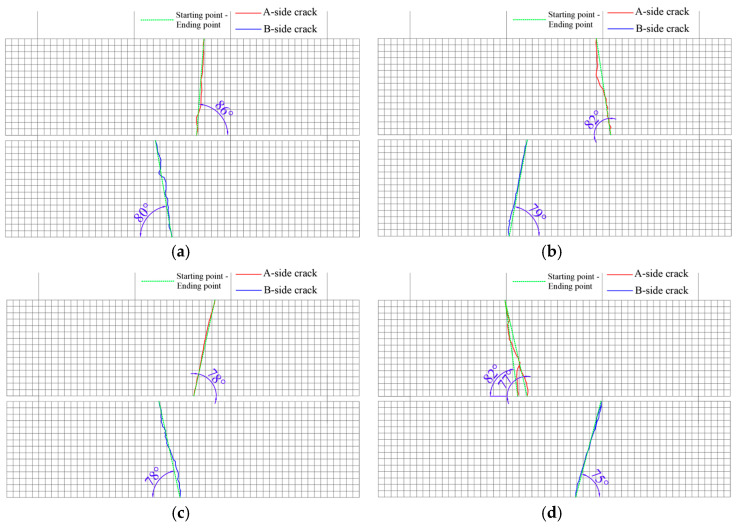
Crack angles of groups 5–8 at a stress level of 0.65. (**a**) C1 = 30%; C2 = 0%; C3 = 10%, (**b**) C1 = 30%; C2 = 20%; C3 = 10%, (**c**) C1 = 30%; C2 = 30%; C3 = 10%, (**d**) C1 = 30%; C2 = 40%; C3 = 10%.

**Figure A16:**
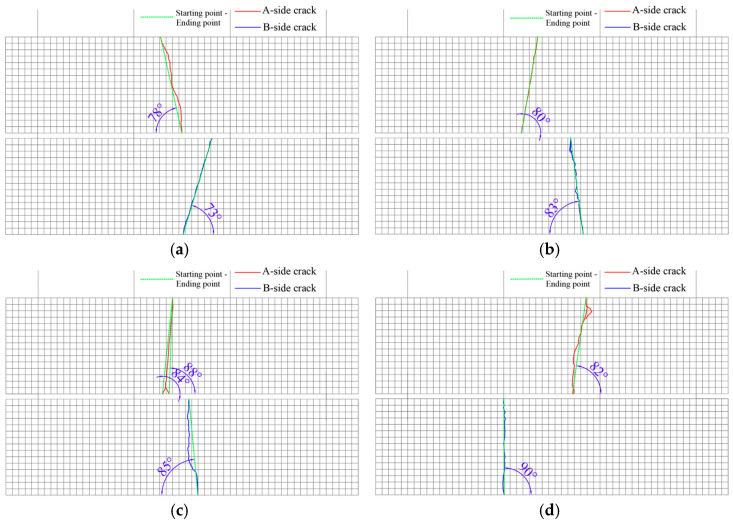
Crack angles of groups 9–12 at a stress level of 0.65. (**a**) C1 = 30%; C2 = 10%; C3 = 0%, (**b**) C1 = 30%; C2 = 10%; C3 = 20%, (**c**) C1 = 30%; C2 = 10%; C3 = 30%, (**d**) C1 = 30%; C2 = 10%; C3 = 40%.

**Figure A17:**
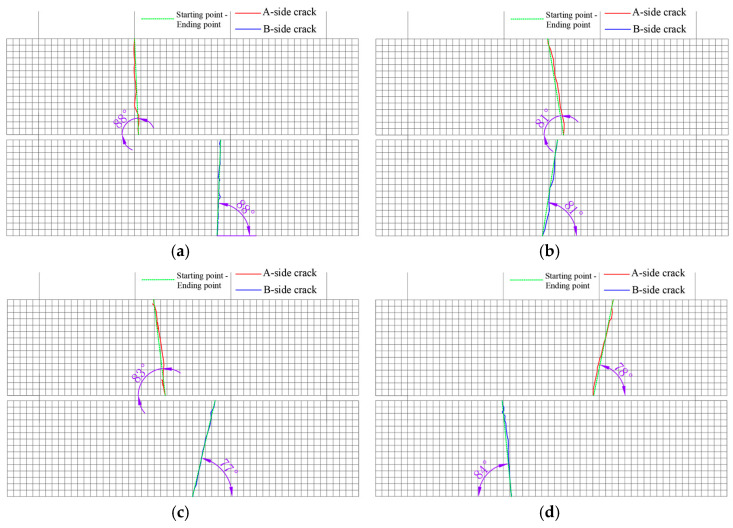
Crack angles of groups 5–8 at a stress level of 0.75. (**a**) C1 = 30%; C2 = 0%; C3 = 10%, (**b**) C1 = 30%; C2 = 20%; C3 = 10%, (**c**) C1 = 30%; C2 = 30%; C3 = 10%, (**d**) C1 = 30%; C2 = 40%; C3 = 10%.

**Figure A18:**
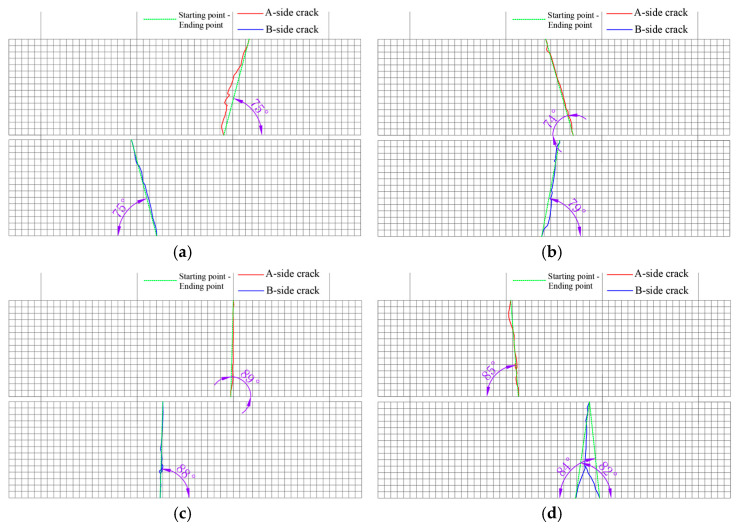
Crack angles of groups 9–12 at a stress level of 0.75. (**a**) C1 = 30%; C2 = 10%; C3 = 0%, (**b**) C1 = 30%; C2 = 10%; C3 = 20%, (**c**) C1 = 30%; C2 = 10%; C3 = 30%, (**d**) C1 = 30%; C2 = 10%; C3 = 40%.

## Data Availability

The original contributions presented in this study are included in the article. Further inquiries can be directed to the corresponding author.
